# Supplementary material for: Alternative female and male developmental trajectories in the dynamic balance of human visual perception
Source: Sci Rep. 2022 Jan 31;12:1674. doi: 10.1038/s41598-022-05620-1 (PMC8803928; doi:10.1038/s41598-022-05620-1)
Supplement: Supplementary file 1 — Supplementary Information. [file 41598_2022_5620_MOESM1_ESM.pdf]

Supplementary information

Alternative female and male developmental trajectories in the dynamic balance of human visual perception

Gergő Ziman[a,b,c,*], Stepan Aleshin[d], Zsolt Unoka[e], Jochen Braun[f], and Ilona Kovács[a,b,g]

[a] Laboratory for Psychological Research, Pazmany Peter Catholic University, Mikszath ter 1, 1088 Budapest, Hungary
[b] Adolescent Development Research Group, Hungarian Academy of Sciences - Pazmany Peter Catholic University, Mikszath ter, 1088 Budapest, Hungary
[c] Doctoral School of Mental Health Sciences, Semmelweis University, Balassa u. 6., 1083 Budapest, Hungary
[d] Institute of Biology (IBIO), Otto-von-Guericke-Universität, Universitätsplatz 2, 39120 Magdeburg, Germany
[e] Department of Psychiatry and Psychotherapy, Semmelweis University, Üllői út 26, 1085 Budapest, Hungary
[f] Cognitive Biology, Otto-von-Guericke Universität, Leipziger Str 44, 39120 Magdeburg, Germany
[g] Institute of Cognitive Neuroscience and Psychology, Research Centre for Natural Sciences, Tudosok krt. 1, 1117 Budapest, Hungary

*corresponding author: Gergő Ziman, ziman.gergo@btk.ppke.hu

Supplementary information

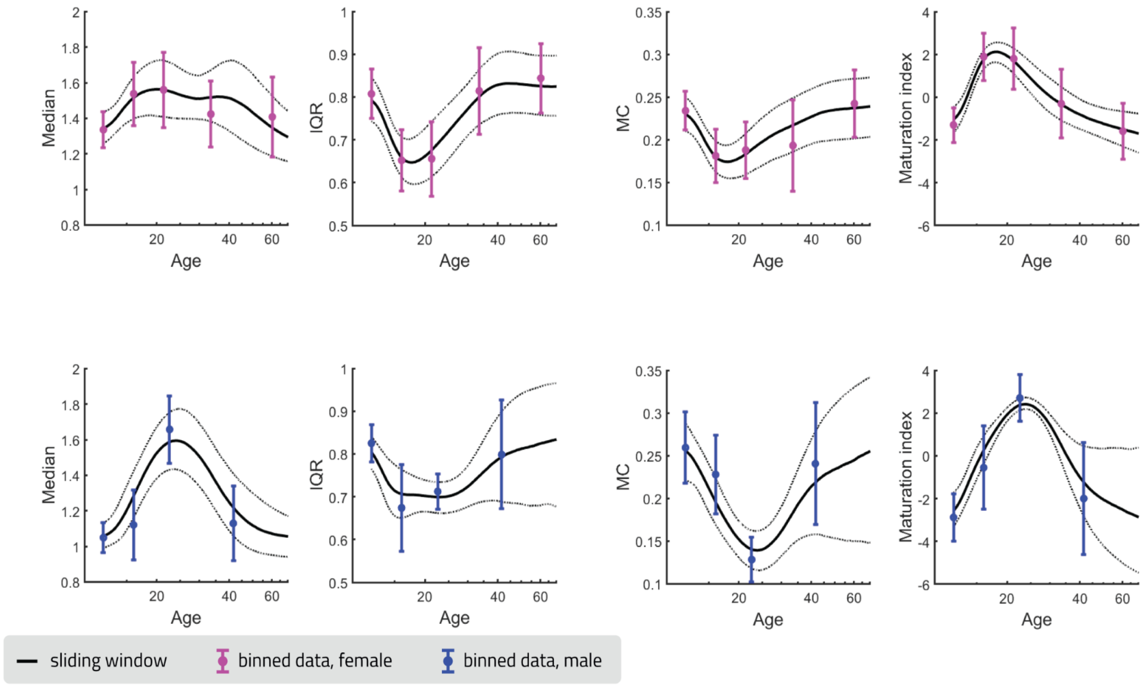

Supplementary figure 1 One-dimensional plots of binocular rivalry observables (as in Fig. 2 a, b) and maturation index (as in Fig. 2 d). Symbols and error bars show mean and standard deviation of measured values within non-overlapping age bins. Smooth curves show the median and standard-deviation of Gaussian-weighted sliding-window averages, obtained over random samples of observers (10000 samples with replacement).

Supplementary information

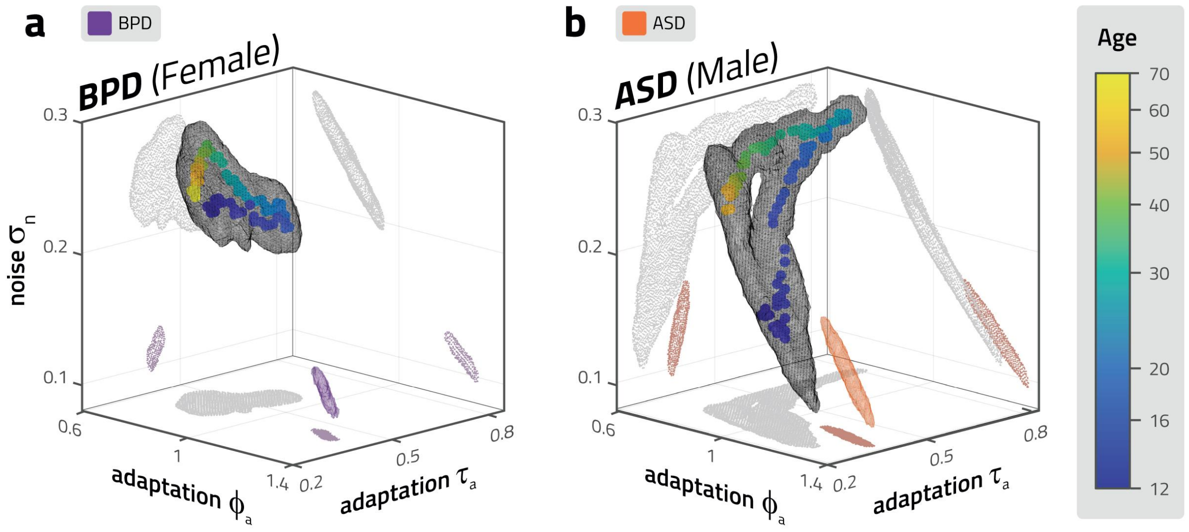

Supplementary figure 2 Multistable perception model parameters reproducing observed results of clinical groups. a, **b**: Combinations of model parameter values reproducing dominance statistics (within a ±5% tolerance range) of clinical observer groups (BPD in purple, ASD in orange) in ϕ_a-τ_a-σ_n subspace, with constant competition ß=3, presented alongside typical developmental and maturational trajectories of the respective sex (same as in Fig 3 c, d).

Supplementary information

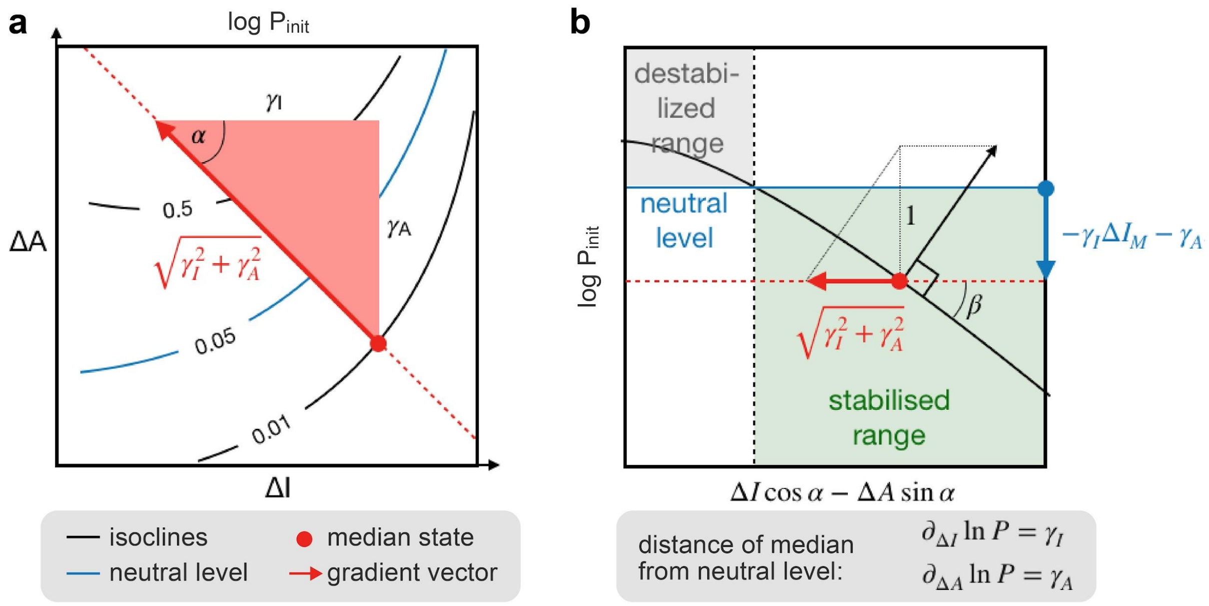

SENSITIVITY	STABILITY	EXPLORATION
length of gradient vector	distance of median from neutral level	direction of gradient vector
$\tan\beta = \sqrt{\gamma_I^2 + \gamma_A^2}$	$-\gamma_I \Delta I_M - \gamma_A \Delta A_M$	$\sin\alpha = \frac{\gamma_A}{\sqrt{\gamma_I^2 + \gamma_A^2}}$

Supplementary figure 3 Mathematical definitions of perceptual parameters. a: The conditional likelihood of reversals as a function of ΔI and Δa values during initiation periods, in the vicinity of the median state (over all periods, red dot). The reversal probability grows exponentially to the direction of the red line. Sensitivity is defined as the length of the gradient vector ∂ logP(ΔI,Δa).Exploration is defined as the direction of the gradient vector, α. **b**: Enlarged cut through the log P(ΔI,Δa) surface in the direction of the the gradient vector (red arrow), showing the neutral level (defined by ΔI=Δa=0, black dashed line) and median level (red dashed line). Stability is defined as the distance between these levels. At positive values of stability (stabilized range), median states lower reversal probability; vice versa for negative values (destabilized range). Auxiliary variables γ_I and γ_A are the partial derivatives $\gamma_I = \partial_{\Delta I} lnP$ and $\gamma_A = \partial_{\Delta A} lnP$.
